# Development of Immunoassay Based on Monoclonal Antibody Reacted with the Neonicotinoid Insecticides Clothianidin and Dinotefuran

**DOI:** 10.3390/s121115858

**Published:** 2012-11-15

**Authors:** Mikiko Uchigashima, Eiki Watanabe, Shigekazu Ito, Seiji Iwasa, Shiro Miyake

**Affiliations:** 1 Research & Development Division, HORIBA, Ltd., Minami-ku, Kyoto 601-8510, Japan; E-Mails: mikiko.uchigashima@horiba.com (M.U.); shigekazu.ito@horiba.com (S.I.); 2 National Institute for Agro-Environmental Sciences, Tsukuba, Ibaraki 305-8604, Japan; E-Mail: eikiw@affrc.go.jp; 3 Department of Materials Science, Toyohashi University of Technology, Toyohashi, Aichi 441-8580, Japan; E-Mail: iwasa@ens.tut.ac.jp; 4 Advanced Scientific Technology & Management Research Institute of Kyoto, Shimogyo-ku, Kyoto 600-8813, Japan; E-Mail: miyake@astem.or.jp

**Keywords:** enzyme-linked immunosorbent assay, ELISA, hapten

## Abstract

Enzyme-linked immunosorbent assay (ELISA) based on a monoclonal antibody (MoAb) was developed for the neonicotinoid insecticide clothianidin. A new clothianidin hapten (3-[5-(3-methyl-2-nitroguanidinomethyl)-1,3-thiazol-2-ylthio] propionic acid) was synthesized and conjugated to keyhole limpet hemocyanin, and was used for monoclonal antibody preparation. The resulting MoAb CTN-16A3-13 was characterized by a direct competitive ELISA (dc-ELISA). The 50% of inhibition concentration value with clothianidin was 4.4 ng/mL, and the working range was 1.5–15 ng/mL. The antibody showed high cross-reactivity (64%) to dinotefuran among the structurally related neonicotinoid insecticides. The recovery examinations of clothianidin for cucumber, tomato and apple showed highly agreement with the spiked concentrations; the recovery rate was between 104% and 124% and the coefficient of variation value was between 1.8% and 15%. Although the recovery rate of the dc-ELISA was slightly higher than that of HPLC analysis, the difference was small enough to accept the dc-ELISA as a useful method for residue analysis of clothianidin in garden crops.

## 1. Introduction

**Figure 1.**
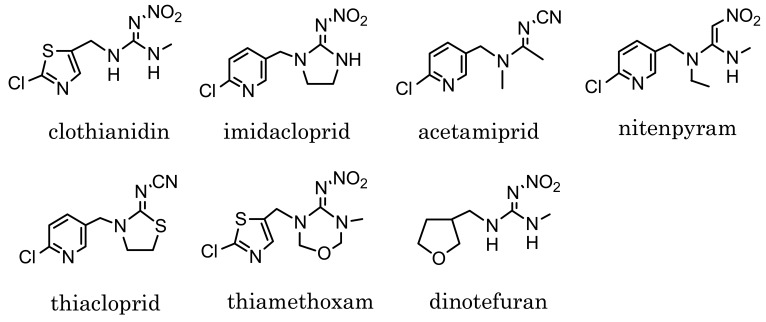
Structure of neonicotinoid insecticides.

Neonicotinoids such as those shown in Figure 1 are relatively new insecticides structurally related each other, and are originated from imidacloprid, which has been put on the market since 1992 [1–3]. Neonicotinoid insecticides are similar to nicotinoids in binding to the nicotinic acetylcholine receptor of insects [4,5]. Because of the high sensitivity of insects and low toxicity to mammals, they have been used worldwide as substitutes of conventional insecticides, e.g., organophosphates, carbamates and pyrethroids [6]. Clothianidin, (*E*)-1-(2-chloro-1,3-thiazol-5-ylmethyl)-3-methyl-2-nitroguanidine, which is one of the neonicotinoid insecticides, is effective against various insects, such as hemiptera, diptera, lepidoptera, coleoptera, thysanoptera and orthoptera species. Easily absorbed and transported into plants, it has been applied for paddy rice and various garden crops after registration in Japan in 2002 [7]. Dinotefuran, 2-methyl-1-nitro-3-(tetrahydrofuran-3-ylmethyl)guanidine, which was registered in 2004 as a neonicotinoid insecticide, is also effective against various insects like clothianidin [8]. At present, dinotefuran is one of the most widely used insecticides for garden crops in Japan.

The maximum residue limits (MRLs) of clothianidin and dinotefuran for garden crops in Japan have been set to 0.2–15 mg/kg and 0.5–25 mg/kg, respectively. The analyses of their residues are conducted by high performance liquid chromatography (HPLC) like other insecticides [9,10]. Although the HPLC method is sufficiently sensitive and accurate for laboratory examination, analyzing residues on-site by HPLC is not practical because it is too complicated, time-consuming and expensive. As an alternative method which can be widely accepted for on-site monitoring, immunoassays have been applied to determine many kinds of agrochemicals [11–18]. They are not only simple, rapid and cost-effective methods compared to HPLC, but also practically sensitive and accurate enough for on-site analysis.

We have developed an immunoassay which is an enzyme-linked immunosorbent assay (ELISA) for clothianidin, using a newly synthesized hapten and a monoclonal antibody (MoAb) prepared against clothianidin in this study. The ELISA showed a sufficient reactivity not only towards clothianidin, but to dinotefuran as well. This paper describes preparation of the MoAb, development of the ELISA using the MoAb, and applicability of the ELISA for clothianidin residue analysis in garden crop samples.

## 2. Experimental Section

### 2.1. Reagents and Apparatus

Clothianidin, imidacloprid, nitenpyram and thiamethoxam standards were purchased from Wako Pure Chemical Industries (Osaka, Japan). Acetamiprid standard was purchased from Kanto Chemicals (Tokyo, Japan). Thiacloprid standard was from Riedel-de Haën (Hannover, Germany). Dinotefuran standard was kindly provided from Mitsui Chemical (Tokyo, Japan). The other chemicals were from Wako Pure Chemical and Tokyo Kasei Kogyo (Tokyo, Japan). Keyhole limpet hemocyanin (KLH) was from Wako Pure Chemical. Horse radish peroxidase (HRP) was purchased from Toyobo (Osaka, Japan). Bovine Serum albumin (BSA) and HAT reagent, polyethylene glycol (PEG) 1500 reagent and Dulbecco's Modified Eagle Medium (DMEM) were from Sigma-Aldrich (St. Louis, MO, USA). HRP labeled anti-mouse IgG was from MP Biomedicals (Solon, OH, USA). Protein G column was from GE Healthcare (Buckinghamshire, England). Freund's complete and incomplete adjuvants were from BD-Diagnostic Systems (Sparks, MD, USA). Fetal bovine serum (FBS) was from Invitrogen (Carlsbad, CA, USA). Microtiter-plate with 96-wells for ELISA and microplate with 96-wells for cell culture were purchased from Thermo Fisher Scientific (New York, NY, USA). Cartridges used for solid-phase extraction (SPE) were Envi-Carb/NH_2_ (500 mg + 500 mg/6 mL; Supelco, Bellefonte, PA, USA). ^1^H-NMR spectra were measured on the Bruker DRX500 spectrometer. Chemical shifts in ^1^H-NMR are described in parts per million downfield from tetramethylsilane as an internal standard (ρ 7.71) in CDCl_3_, unless otherwise noted. ELISAs were measured on the MPR-01 microplate reader from Horiba (Kyoto, Japan).

### 2.2. Hapten Synthesis

**Scheme 1.**
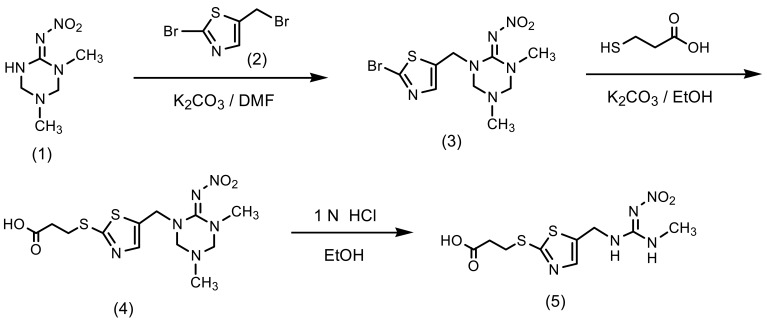
Synthetic scheme of clothianidin hapten.

The synthetic pathway to a clothianidin hapten is outlined in Scheme 1. To a solution of 1-methyl-3-nitroguanidine (6.0 g, 50 mmol) in ethanol (110 mL) was added 38% formaldehyde solution (12.6 g, 154 mmol) and 40% methylamine solution (6.0 g, 77 mmol). The mixture was stirred at 50 °C for 4 h and then concentrated. The resulting solid was re-crystalized from ethyl acetate-methanol (4/1, v/v) to give 5.0 g (57% yield) of 1,5-dimethyl-2-nitroiminohexahydro- 1,3,5-triazine (1) as a white solid (mp: 134–135 °C): ^1^H-NMR (DMSO-d_6_) δ; 2.43 (3H, s, CH_3_), 2.84 (3H, s, CH_3_), 4.22 (2H, s, CH_2_), 4.25 (2H, s, CH_2_), 9.24 (1H, s, NH). To a solution of 2-amino-5-methylthiazole (12.0 g, 110 mmol) in 48% hydrobromic acid (71 g, 420 mmol) and water (14 mL) was added sodium nitrite (8.2 g, 120 mmol), and then cupper powder (0.42 g) at −4 °C (ice-salt bath). After removing the ice-salt bath, the mixture was stirred at 50 °C for 15 min until bubbles ceased. To the reaction mixture was added water (120 mL) and the product was extracted with toluene. The toluene layer was washed with water, dried over anhydrous magnesium sulfate, filtered and concentrated to give 8.0 g (43% yield) of 2-bromo-5-methylthiazole as a pale yellow oil. To a mixture of the thiazole (8.0 g, 45 mmol) and *N*-bromosuccinimide (8.8 g, 50 mmol) in carbon teterachloride (90 mL) was added benzoyl peroxide (0.36 g, 1.5 mmol). The mixture was then refluxed for 50 min, cooled to room temperature, filtered and concentrated. The residue was purified by flash column chromatography on silica gel eluted with n-hexane/dichloromethane (6/1→2/3, v/v) to give 5.7 g (44% yield) of 2-bromo-5-bromomethylthiazole (2) as oil.

A mixture of 1,5-dimethyl-2-nitroiminohexahydro-1,3,5-triazine (1) (3.4 g, 20 mmol), potassium carbonate (3.0 g, 22 mmol) and 2-bromo-5-bromomethylthiazole (2) (5.6 g, 22 mmol) in acetonitrile (65 mL) was refluxed for 3 h. The reaction mixture was concentrated under reduced pressure. To the residue was added methanol (30 mL) and filtered. The filtrate was concentrated to give 1-(2-bromothiazol-5-ylmethyl)-2-nitroimino-3,5-dimethylhexahydro-1,3,5-triazine (3) as a brown oil (1.8 g, 26% yield). ^1^H-NMR (CD_3_OD) δ; 2.06 (3H, s, CH_3_), 2.84 (3H, s, CH_3_), 4.34 (2H, s, CH_2_), 4.39 (2H, s, CH_2_), 4.70 (2H, s, CH_2_), 7.65 (1H, s, CH): To a solution of the triazine (3) (1.2 g, 3.4 mmol) in ethanol (10 mL) was added 3-mercaptopropionic acid (0.43 g, 4 mmol) and potassium carbonate (1.1 g, 8 mmol). The mixture was stirred for 10 h under reflux, and concentrated under reduced pressure. The residue was acidified at pH 3 by adding water (10 mL) and 1N hydrochloric acid, and the product was extracted with ethyl acetate (30 mL). The organic layer was washed with water, dried over anhydrous magnesium sulfate, filtered and concentrated to give 3-[5-(3,5-dimethyl-6-nitroimino- hexahydro-1,3,5-triazin-1-ylmethyl)thiazol-2-ylthio]propionic acid (**4**) as a brown oil (0.7 g, 68% yield). The propionic acid (**4**) (0.45 g, 1.1 mmol) and 1N hydrochloric acid (6 mL) in ethanol (10 mL) was stirred at 40 °C for 5 h and concentrated under reduced pressure. The residue was purified by flash column chromatography on silica gel eluted with ethyl acetate/methanol (9.5/0.5, v/v) to give 3-[5-(3-methyl-2-nitroguanidinomethyl)-1,3-thiazol-2-ylthio]propionic acid (hapten) (5) as a yellow oil (0.19 g, 55% yield). ^1^H-NMR (CD_3_OD) δ; 2.76 (2H, t, *J* = 5.0 Hz, CH_2_), 2.90 (3H, s, CH_3_), 3.39 (2H, t, *J* = 7.5 Hz, CH_2_), 4.61 (2H, s, CH_2_), 7.58 (1H, s, CH).

### 2.3. Preparation of Hapten—Protein Conjugates

Clothianidin hapten was conjugated with KLH to immunize to mice, with BSA to constitute a direct-bind ELISA (db-ELISA) and an indirect competitive ELISA (ic-ELISA), and with HRP to constitute a direct-competitive ELISA (dc-ELISA), by the activated ester method as previously described [19]. The hapten (20 μmol) was dissolved in 1 mL of dried dimethyl sulfoxide. N-hydroxysuccinimide (40 μmol) and 1-ethyl-3-(3-dimethylamiopropyl) carbodiimide hydrochloride (40 μmol) were added in the solution. The solution was stirred at room temperature for 1.5 h to esterify carboxylic acid of the hapten and the succinimide. Each 200 μL of the stirred solution was added to the protein (20 mg) dissolved in 1 mL of borate buffered saline (100 mmol/L sodium borate, 150 mmol/L NaCl, pH 8.0). Each of the mixture was gently stirred at room temperature for 1.5 h to combine the activated carboxyl group of the hapten with amino group of L-lysine residues in the proteins. The combined hapten-KLH and the hapten-BSA conjugates were dialyzed against phosphate buffered saline (PBS; 10 mmol/L sodium phosphate, 150 mmol/L NaCl, pH 7.2) at 4 °C for 4 days to remove the uncombined chemicals. The hapten-HRP conjugate was also dialyzed, and was further purified through the gel filtration column.

### 2.4. MoAb Preparation

A MoAb was prepared using the previously described procedure with a slight modification [20]. Seven weeks female Balb/c mice from Nippon SLC (Shizuoka, Japan) were intraperitoneally immunized with 100 μL of the hapten-KLH conjugate (100 μg per a mouse) after it had been emulsified with an equal volume of Freund's complete adjuvant. Booster immunization (25 μg per a mouse) was performed 4 times using the emulsion with Freund's incomplete adjuvant at intervals of 2 weeks. After the third immunization, 50 μL of the blood was taken from the tail vein, and the sera were prepared. On the third days after the last immunization, spleen cells from the mice (1.4 × 10^7^ cells/mouse) were fused with P3-X63-AG8.653 myeloma cells (2.0 × 10^6^ cells/mouse) by using PEG 1,500 reagent. The fused cells were suspended at 2.5 × 10^6^ cells/mL as spleen cells in DMEM medium modified with 10% FBS and with HAT reagent (hypoxanthine: 100 μM, aminopterin: 0.4 μM, thymidine: 16 μM). Each 100 μL of the cell suspension was transferred to the wells of a 96-well microplate. The microplate was incubated at 37 °C for 10 days in 5% CO_2_. After confirming that the growing hybridoma had formed a colony, each of secreted antibody in the cultured fluids was screened on the basis of reactivity with the hapten-BSA conjugate by the db-ELISA. Fluids in the positive wells were secondary screened on the basis of reactivity with clothianidin by the ic-ELISA. Each hybridoma grown in the positive wells was cloned by limiting dilution technique (2 times), and the representative cell clone was used for preparation of the MoAb.

For MoAb preparation, Balb/c mice were pretreated by intraperitoneal injection with 0.5 mL of pristane, and 1 week after the pretreatment, the mice were inoculated with 2 × 10^7^ viable cells. Seven to 10 days after the inoculation, ascite fluids were collected from the mice, and the MoAb in the fluid was purified with a protein G column. In brief, 0.5 mL of the pooled ascite fluid was centrifuged at 9,000 rpm for 5 min, and the supernatant was mixed with equal volume of 50 mM sodium phosphate buffer (pH 7.0). The mixture was applied to the protein G column in which the gel volume was 1 mL. After the application, the column was washed with 10 mL of the 50 mM phosphate buffer (pH 7.0). The MoAb was eluted with 1 mL of 0.1 M glycine-HCl buffer (pH 3.0), and immediately adjusted to pH 7.0 with 1.0 M Tris-HCl buffer (pH 9.0). The MoAb concentration was determined from the extinction coefficient (1.4 for 1.0 mg/mL of IgG).

### 2.5. db-ELISA and ic-ELISA

A db-ELISA and an ic-ELISA were constituted using the previously described procedure with a slight modification [20]. For both of the ELISA constitutions, 100 μL of the hapten-BSA conjugate (5.0 μg/mL) dissolved in PBS was initially added to each well of 96-well microtiter plate. The conjugate was coated to the well by incubation at 4 °C for overnight. After the coated well was washed 3 times with PBS, the well was blocked with 300 μL of blocking reagent (PBS-B) which was PBS modified with 10% Block Ace from Dainippon Sumitomo Pharma (Osaka, Japan), by incubation at 25 °C for 1 h. The blocked well was washed three times with PBS.

For a db-ELISA, each 50 μL of cultured fluid of the hybridoma diluted with PBS-B was added to the blocked well. The microtiter plate was incubated at 25 °C for 1 h. After the plate was washed three times with PBS, 100 μL of HRP-labeled goat anti-mouse IgG antibody diluted 8,000-fold with PBS-B was added to each well. The plate was incubated at 25 °C for 1 h and then washed three times with PBS. A hundred μL of HRP substrate solution (2 mg/mL of 3,3′,5,5′-tetramethylbenzidine and 0.006% H_2_O_2_ dissolved in 100 mM sodium acetate buffer, pH 5.5) was added to each well, and the plate was incubated at 25 °C for 10 minutes to develop color reaction. The reaction was stopped by addition of 100 μL of 500 mM sulfuric acid. The absorbance was measured at 450 nm in the microplate reader.

For an ic-ELISA, clothianidin was dissolved and serially diluted with methanol. The diluents were further diluted to 10 folds with PBS-B: the prepared concentrations were 100–100,000 ng/mL in 10% methanol. The cultured fluid was diluted 2-fold with PBS-B whereby 50% of maximum absorbance was developed by the above db-ELISA. Each 50 μL of the clothianidin solution was added to the above blocked wells, and then 50 μL of the diluted cultured fluid was immediately mixed in the wells. The plate was incubated at 25 °C for 1 h. The following steps were taken as described in the above db-ELISA.

### 2.6. dc-ELISA

A dc-ELISA was constituted using the previously described procedure with a slight modification [19]. A rabbit anti-mouse IgG antibody (100 μL, 10 μg/mL) dissolved in PBS was added to each well of a 96-well microtiter plate. The plate was incubated at 4 °C overnight. After the antibody coated plate was washed three times with PBS, the wells were blocked with BSA by addition of 300 μL of 0.4% BSA dissolved in PBS. The plate was incubated at 25 °C for 1 h. A hundred μL of purified MoAb (2 μg/mL) dissolved in PBS modified with 0.2% BSA (PBS-BSA) was added to each well, and the MoAb was bound to the coated antibody based on antigen-antibody reaction by incubation at 25 °C for 1 h.

Clothianidin was dissolved and serially diluted with methanol. The diluents were further diluted 10-fold with PBS-BSA: the prepared concentrations were 0.1–200 ng/mL. A hapten-HRP conjugate was diluted to the adequate concentration (14 ng/mL) with PBS-BSA, so that the maximum absorbance showed around 1.2 at 450 nm by this dc-ELISA. Each of the clothianidin solutions or sample solutions prepared from garden crops was mixed with an equal volume of the hapten-HRP conjugate solution (28 ng/mL), and 100 μL of the mixture was added to the above well. The plate was incubated at 25 °C for 1 h. After the plate was washed three times with PBS, color development steps were taken as described in the above db-ELISA. The working range of the dc-ELISA was defined as the clothianidin concentrations between 20% of inhibition concentration (IC_20_) value and 80% of inhibition concentration (IC_80_) value from the mean value of four replicates. The detection limit and the quantification limit were not defined because such values for manual methods depend on experimenters.

### 2.7. HPLC Analysis

To examine correlation between the developed dc-ELISA and the conventional HPLC analysis, a HPLC system consisted of an Agilent 1100 series instrument equipped with a quaternary pump, an autosampler, a column oven and a diode array detector was used. The wavelength for clothianidin detection was 270 nm. The column was a SunFire C_18_ reversed-phase column (5 μm, 4.6 i.d. × 250 mm; Waters, Milford, MA, USA) with a guard column (5 μm, 4.6 i.d. × 20 mm). The mobile phase was acetonitrile/water (25/75, v/v). The flow rate was 0.85 mL/min, the column oven temperature was 40 °C, and the injection volume was 20 μL.

### 2.8. Treatment of Garden Crop Samples

A garden crop samples for dc-ELISA was treated on the basis of the previously described procedure [19]. Cucumber, tomato, and apple were collected from a vegetable shop selling pesticide-free garden crops. Each sample was homogenized, and the homogenates (5 g) was put in a 50 mL plastic tube. Twenty five mL of methanol was added to each tube, and the tube was hermetically sealed with the screw cap. It was vigorously shaken for 30 minutes to extract the garden crop matrices and/or spiked clothianidin as described below. The extract was filtered through a filter paper, and 1 mL of the filtrate was diluted with 7.5 mL of distilled water. The diluted sample was used for the dc-ELISA; the methanol concentration was equivalent to 10%. Further dilutions of the diluted sample were carried out with water/methanol (9/1, v/v; 10% methanol). To determine clothianidin spiked in garden crop extract, 100 μL of clothianidin dissolved in methanol was added to the 5.9 mL of the above methanol extract; the concentrations were equivalent to 0.077–0.77 mg/kg in garden crops. Separately, 100 μL of clothianidin was spiked to the above homogenized crop samples and stood for 30 min for recovery examination; the concentrations were equivalent to 0.1–0.6 mg/kg.

A garden crop sample for HPLC analysis was treated on basis of the procedure by Ministry of Health, Labour and Welfare in Japan [20]. The homogenized sample (20 g) spiked with clothianidin was extracted with 50 mL of acetonitrile for 3 min using a homogenizer (Polytron PT2100; Kinematica AG, Lucerne, Switzerland). After the extraction, the mixture was filtered through a funnel by suction. The residue was then re-treated with 20 mL of acetonitrile. Both extracts were accurately made up to 100 mL with acetonitrile in a volumetric flask, and then 20 mL aliquots of the extract, equivalent to 4 g of sample, was mixed with 10 g of sodium chloride and 20 mL of 0.5 M phosphate buffer (pH 7.0). The mixture was vigorously shaken for 10 min, and stood for about 10 min. After the aqueous phase was discarded, the acetonitrile phase was dried, filtered, and then concentrated. After the residue was reconstituted in 2 mL of acetonitrile/toluene (3/1, v/v) and the solution was applied to an Envi-Carb/NH_2_ SPE cartridge preconditioned with 10 mL of acetonitrile/toluene (3/1, v/v). The retained clothianidin was eluted with 20 mL of acetonitrile/toluene (3/1, v/v). The eluate was concentrated, and then the residue was reconstituted in 10 mL of acetone. The solution was concentrated, to the residue 5 mL of acetone was added, and then the acetone was evaporated under a gentle nitrogen stream at 50 °C. The residue was reconstituted in 1 mL of acetonitrile/water (25/75, v/v) and syringed into an autosampler vial of the HPLC after filtered using a 0.45 nm PTFE filter (Millipore, Billerica, MA, USA).

## 3. Results and Discussion

### 3.1. Preparation of an Anti-Clothianidin MoAb

Designing the hapten is one of the most important steps in preparation of a MoAb which reacts with the target pesticides [19,21–25]. Four compounds among the neonicotinoid insecticides shown in Figure 1 have a six-membered pyridine ring in their structure. The others have a five-membered ring instead of pyridine: thiazolyl ring in clothianidin and thiamethoxam, and tetrahydrofuran ring in dinotefuran. In the case of designing a clothianidin hapten, the neonicotinoids with pyridine ring are expected to show negligible cross-reactivity with a clothianidin-specific MoAb, because of six-membered ring which is likely to cause steric hindrance. On the other hand, thiamethoxam and dinotefuran might have the cross-reactivity with the prepared MoAb. The structure of the clothianidin hapten we selected is shown in Scheme 1. In this structure, the possibility of cross-reaction with thiamethoxam is considered to be low for the prepared MoAb. However, the possibility of cross-reaction with dinotefuran cannot be ignored due to 3-methyl-2-nitroguanidinyl moiety which is commonly included in the structure of clothianidin and dinotefuran. Finally, we decided to prepare a MoAb which would be specific to both clothianidin and dinotefuran.

The reactivity of anti-sera from immunized mice was examined in db-ELISA firstly. The titers of sera from 4 mice were between 1 × 10^5^ and 2 × 10^5^. The results showed that the sera titers were high against the hapten bound to BSA. Reactivity with free clothianidin was secondary examined in ic-ELISA. The sera reacted with clothianidin in the concentration range of 2.5–10 μg/mL. On the other hand, the reactivity range of target MoAb should be 1–10 ng/mL as clothianidin concentration. This means required reactivity of MoAb is 1,000 times higher than that of sera. Cell fusion technique was therefore applied to pick up a candidate cell clone among large numbers of hybridomas, which may secret highly reactive MoAb. Among wells on 16 microplates, on which colonies of incubated hybridomas were formed, 20 wells were positive when cut off value was Abs. 0.4 in the db-ELISA. Cultured fluids of three wells showed 50% of inhibition concentration (IC_50_) values of <0.63 μg/mL in the ic-ELISA. Cells in the three wells were cloned by limiting dilution technique, and a cell clone secreting MoAb CTN-16A3-13 was finally selected to prepare the highest reactive MoAb with clothianidin (data not shown). The MoAb CTN-16A3-13 was purified and was used to constitute dc-ELISA.

### 3.2. dc-ELISA Constitution Based on MoAb

**Figure 2.**
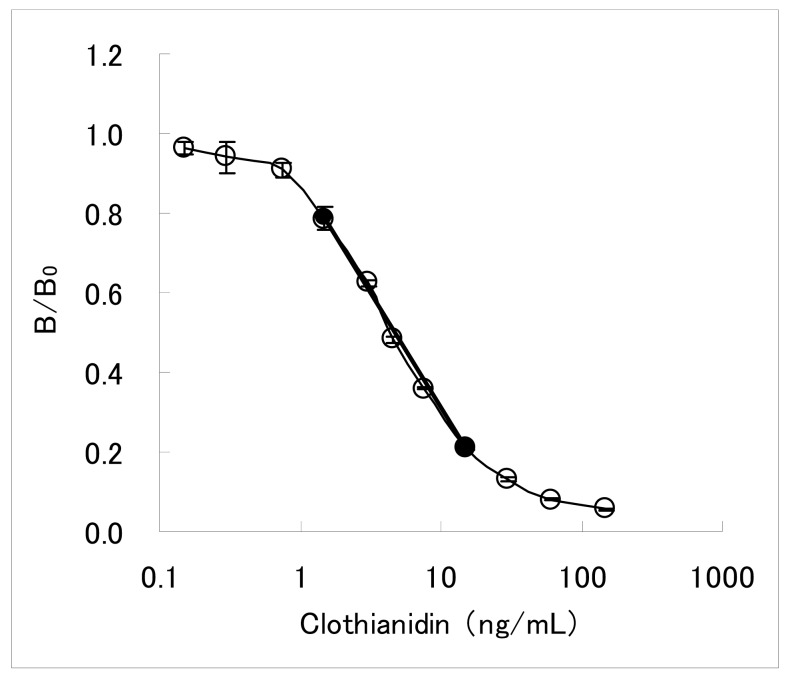
Inhibition curve of clothianidin in the dc-ELISA. Thin line with white circle (○) shows inhibition curve, and thick straight line between the IC_20_ and IC_80_ values (●) shows 2-point standard curve. Each point represents the mean value of 4 replicates and error bars indicate standard deviations. B/B_0_ shows ratio between absorbance for clothianidin concentration (B) and maximum absorbance (B_0_) at 450 nm.

The dc-ELISA is a simpler assay than the ic-ELISA described above, requiring less assay steps. It has been therefore adopted for developing commercially available test kits for agrochemicals immunoassay [26–31]. The dc-ELISA targeted clothianidin residue analysis in garden crops has been also constituted. Concentrations of the MoAb CTN-16A3-13 and the hapten-HRP conjugate were 2.0 μg/mL and 0.014 μg/mL, respectively. Under this condition, the inhibition curve with working range of 1.5–15 ng/mL was obtained as shown in Figure 2. Here, the working range is defined as the concentration range from IC_20_ value to IC_80_ value. The IC_50_ value is 4.4 ng/mL. The inhibition curve in the working range seemed to be almost linear, and a linear line calculated from IC_20_ and IC_80_ values (y = −0.58 log_10_X + 0.89) was considered to be applicable as a 2-point standard curve. In our experience, dc-ELISAs for pesticide residue analyses typically needed 51-fold dilution of the samples [19]. For most garden crops of which MRLs is from 0.2 to 15 mg/kg for clothianidin, the sensitivity of the developed dc-ELISA was considered to be sufficient for analyzing the residual clothianidin with such dilution process.

The cross-reactivity is also important for the dc-ELISA. As already mentioned in the previous section, the MoAb CTN-16A3-13 prepared for dc-ELISA is expected to react with dinotefuran not only with clothianidin. Table 1 summarizes the cross-reactivity of the MoAb. The MoAb exactly reacted with dinotefuran at the cross-reactivity of 64%. It was found that the MoAb CTN-16A3-13 is highly specific antibody to clothianidin and dinotefuran. The MoAb did not react with thiamethoxam which is a precursor of clothianidin in insects and plants [32]. Thiamethoxam has similar structure with clothianidin except for the ring structure in the nitroguanidinyl moiety. This result suggests that the MoAb specifically recognizes the 3-methyl-2-nitroguanidinyl moiety of clothianidin and dinotefuran. On the other hand, the MoAb CTN-16A3-13 did not clearly distinguish the thiazolyl ring in clothianidin from the tetrahydrofuran ring in dinotefuran. The designed hapten for preparing the MoAb is elongated with a linker at the chlorine position in the thiazolyl ring. One of the possible reasons for the high cross-reactivity with dinotefuran is that the thiazolyl ring is an anchor site which generally shows weak antibody recognition. In addition, the tetrahydrofuran ring of dinotefuran is considered to be soft structure and easy to fit to a binding site of the antibody. The other neonicotinoid insecticides, i.e., imidacloprid, acetamiprid, nitenpyram and thiacloprid, showed no reactivity with the MoAb although they have similar structures as clothianidin. The result is expected from their structures which include six-membered ring without 3-methyl-2-nitroguanidinyl moiety.

As described above, the dc-ELISA based on the MoAb CTN-16A3-13 showed sufficient sensitivity and specificity to clothianidin and dinotefuran, against the MRLs in garden crops which are mainly set 0.2–15 mg/kg and 0.5–25 mg/kg respectively. The dc-ELISA using the MoAb CTN-16A3-13 cannot distinguish clothianidin and dinotefuran. However, the dc-ELISA is considered to be practically applicable for monitoring both clothianidin and dinotefuran, because these insecticides in the same group are seldom applied in the field at the same time, having similar function mechanism and effect. To identify the insecticide type precisely, other analytical methods, such as HPLC, shall be used.

### 3.3. Determination of Clothianidin in Garden Crops

**Figure 3.**
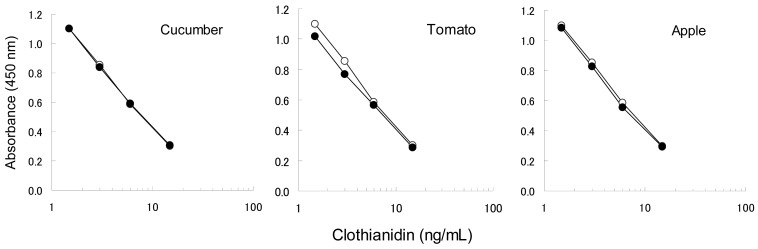
Influence of matrix substances from garden crops in the dc-ELISA. Line with white circle (○) shows inhibition curve between the IC_20_ and IC_80_ values for clothianidin standards in 10% methanol. Line with black circle (●) shows the corresponding inhibition curve for clothianidin in each garden crop extract diluted to 10% methanol.

To confirm the applicability of the dc-ELISA based on the MoAb CTN-16A3-13, the influence of matrix substances from garden crops should be examined. The matrix substances are considered to mainly affect the binding ability of the MoAb with the hapten-HRP conjugate, rather than the insecticides themselves which have low molecular weights. In addition, clothianidin and dinotefuran have similar chemical properties each other. Clothianidin was therefore selected as the representative to be examined here. Four concentrations of clothianidin (1.5, 3.0, 6.0 and 15 ng/mL) spiked in garden crop extracts were compared to the relevant controls (10% methanol with clothianidin) as shown in Figure 3, in order to confirm the influence of matrix from cucumber, tomato and apple. Each clothianidin concentrations were equivalent to 0.077, 0.15, 0.31 and 0.77 mg/kg in the garden crops, which was selected based on the target MRLs, 0.2–15 mg/kg. The results from the extracts were agreed well with the control results for cucumber and apple, while a slight interference was recognized in the case of tomato. Although the interference on tomato resulted in 20%–30% higher than expected, it is acceptable level to apply the dc-ELISA. The dc-ELISA also showed applicable results for dinotefuran as well as clothianidin (data not shown).

Recovery examination was carried out to confirm the extraction efficiency for cucumber, tomato and apple. Clothianidin was spiked at 0.10, 0.25 and 0.60 mg/kg into each crop homogenate, and then each homogenate was stood for 30 minutes. The homogenates were treated under the same condition as that in the examination of matrix interference. As summarized in Table 2, the recovery of cucumber and apple was agreed well with the spiked concentration; the recovery rates were 104–110%. On the other hand, the recovery of tomato was slightly higher but within acceptable range; the recovery rates were 109–124%. The repeatability also seemed good for this examination with three replicates; the coefficient of variation (CV) was in the range of 1.8–14.5%. The results suggest that the dc-ELISA is capable of quantitatively determining the residual clothianidin in the garden crops. The recovery rates of over 100%, especially in tomato, may be derived from the matrix influence, as expected from the results of matrix interference examination. The dc-ELISA also showed applicable recovery results for dinotefuran as well as clothianidin (data not shown).

The recovery results of the conventional HPLC analysis by using clothianidin spiked cucumber and tomato samples is summarized in Table 3. The HPLC analysis showed comparatively low recovery rates (72%–90%), though the dc-ELISA showed slightly higher recovery rate than 100% as already discussed. Such low rates of the HPLC analysis were possibly caused by the loss of clothianidin during the purification processes. The dc-ELISA might give higher values as residual concentrations when compared with the HPLC analysis; however, the dc-ELISA is considered to have sufficient applicability as good as the HPLC analysis.

## 4. Conclusions/Outlook

A hapten for clothianidin which belongs to the neonicotinoid insecticide family was synthesized, and a monoclonal antibody for it was prepared. The dc-ELISA based on the antibody had high affinity to clothianidin (IC_50_ = 4.4 ng/mL) and dinotefuran (64% cross-reactivity). The examinations for cucumber, tomato and apple supported that the dc-ELISA could determine clothianidin in garden crops. The dc-ELISA for clothianidin has been produced as a test kit based on this study. Applicability of the kit will be evaluated in near future. The same dc-ELISA was also expected to be applicable to dinotefuran, which has quite similar physicochemical properties as clothianidin. The recovery results for dinotefuran suggested its applicability, although no actual data was shown in this paper. A dinotefuran test kit has been already produced and reported to have applicability to garden crops [29,30]. Thus, the developed dc-ELISA is expected to be useful for residue analysis of clothianidin and dinotefuran in garden crops. This study was approved by the Bio-management Committee, in-house committee in HORIBA, Ltd., and carried out according to the guidelines of the committee.

## Figures and Tables

**Table 1.** Cross-reactivity of MoAb CTN-16A3-13 with neonicotinoid insecticides in the dc-ELISA.

**Insecticide**	**IC_50_(ng/mL)**	**Cross-reactivity (%)**^a^
**clothianidin**	4.4	100
**dinotefuran**	6.9	64
**acetamiprid**	>5,000	<0.1
**imidacloprid**	>5,000	<0.1
**thiacloprid**	>5,000	<0.1
**nitenpyram**	>5,000	<0.1
**thiamethoxam**	>5,000	<0.1

a Cross-reactivity (%) = (IC_50_ value of clothianidin/IC_50_ value of test insecticides) × 100; Mean values of three replicates are shown.

**Table 2.** Recovery rates of clothianidin spiked into garden crops in the dc-ELISA.

**Garden crop**	**Spiked (ng/g)**	**Detected (ng/g)**^a^	**Recovery (%)**	**CV (%)**
**cucumber**	600	658	110	1.8
	250	260	104	5.7
	100	109	109	11.9

**tomato**	600	655	109	1.4
	250	286	114	3.6
	100	124	124	8.3

**apple**	600	628	105	2.4
	250	265	106	5.1
	100	105	105	14.5

a Mean values of three replicates are shown.

**Table 3.** Recovery rates of clothianidin spiked into garden crops in the HPLC analysis.

**Garden crop**	**spiked (ng/g)**	**detected (ng/g)**^a^	**recovery (%)**	**CV (%)**
**cucumber**	600	480	79.9	8.6
	250	210	84.1	1.9
	100	78	77.5	10.7

**tomato**	600	500	83.0	1.7
	250	210	83.9	2.0
	100	88	88.3	2.7

a Mean values of three replicates are shown.
